# Quality of YouTube videos for patient education on how to use asthma inhalers

**DOI:** 10.1186/1939-4551-8-S1-A221

**Published:** 2015-04-08

**Authors:** Smita Joshi, Ves Dimov

**Affiliations:** 1 Weill Cornell Medical College, USA; 2 University of Chicago, USA

## Background

Inhalers are crucial to the delivery of asthma medications, however their effectiveness is dependent on proper inhaler technique. This study evaluates YouTube videos on their content and ability to inform viewers on how to effectively use an asthma inhaler.

## Methods

The website YouTube.com was queried for the search phrase “how to use asthma inhaler”. The resulting videos were assessed for duration, number of views, number of likes and dislikes, source of video, and content. Content was analyzed for proper inhaler usage. Specifically, videos were assessed whether they discussed the following 9 important steps of inhaler use: removing the cap, priming the inhaler, shaking the inhaler, breathing out before inhaler use, dispensing the medication, taking a deep/slow breath (or several breaths in the pediatric patient using a spacer), holding one’s breath (unless patient took several breaths with a spacer), waiting before taking a repeat dose, and rinsing the mouth after inhaler use.

## Results

The search phrase returned 12,400 videos that were sorted by the default filter of “relevance”. The 20 videos on the first page of the results were analyzed since these are the videos patients are most likely to view by visitor statistics. On average, the videos were 2 minutes and 33 seconds long, with 31,589 views, 47 likes and 5 dislikes. Eight videos were from health care organizations, 5 from a professional society, 2 from health care professionals, and 5 from nonprofessional educational groups. Only 15% of YouTube videos (3 videos) discussed all 9 steps of correct asthma inhaler use. The 3 videos were from health care organizations. The most steps were discussed by videos from health care organizations (average 7.25 steps) and professional societies (average 7 steps). Videos from nonprofessional educational groups (average 4.6 steps) and health care professionals (average 4.5 steps) discussed the least number of steps. Only 50% of videos (10 videos) discussed the use of a spacer.

## Conclusions

The quality of YouTube videos on asthma inhaler use varies considerably. In our analysis, only 15% of videos discussed all steps of correct asthma inhaler use. Videos from health care organizations and medical societies were more comprehensive than those from nonprofessional educational groups and health care professionals not posting on behalf of a medical organization. There is a need for more reliable and accurate patient education videos on YouTube.

